# Investigation of Structural Alterations in Inherited Retinal Diseases: A Quantitative SD-OCT-Analysis of Retinal Layer Thicknesses in Light of Underlying Genetic Mutations

**DOI:** 10.3390/ijms232416007

**Published:** 2022-12-16

**Authors:** Julia Gersch, Katerina Hufendiek, Julien Delarocque, Carsten Framme, Christina Jacobsen, Heidi Stöhr, Ulrich Kellner, Karsten Hufendiek

**Affiliations:** 1 University Eye Hospital, Hannover Medical School, 30625 Hannover, Germany; 2 Clinic for Horses, University Veterinary Medicine Hannover, Foundation, 30559 Hannover, Germany; 3 Institute of Human Genetics, University of Regensburg, Franz-Josef-Strauss-Allee 11, 93053 Regensburg, Germany; 4 Center for Rare Retinal Diseases, AugenZentrum Siegburg, MVZ Augenärztliches Diagnostik- und Therapiecentrum Siegburg GmbH, Europaplatz 3, 53721 Siegburg, Germany; 5 RetinaScience, P.O. Box 301212, 53192 Bonn, Germany

**Keywords:** inherited retinal diseases, SD-OCT, biomarkers

## Abstract

Inherited retinal diseases can result from various genetic defects and are one of the leading causes for blindness in the working-age population. The present study aims to provide a comprehensive description of changes in retinal structure associated with phenotypic disease entities and underlying genetic mutations. Full macular spectral domain optical coherence tomography scans were obtained and manually segmented in 16 patients with retinitis pigmentosa, 7 patients with cone–rod dystrophy, and 7 patients with Stargardt disease, as well as 23 age- and sex-matched controls without retinal disease, to assess retinal layer thicknesses. As indicated by generalized least squares models, all IRDs were associated with retinal thinning (*p* < 0.001), especially of the outer nuclear layer (ONL, *p* < 0.001). Except for the retinal nerve fiber layer, such thinning was associated with a reduced visual acuity (*p* < 0.001). These advances in our understanding of ultrastructural retinal changes are important for the development of gene-, cell-, and optogenetic therapy. Longitudinal studies are warranted to describe the temporal component of those changes.

## 1. Introduction

Inherited retinal diseases (IRD) are one of the leading causes for blindness in the working-age population [1]. In several industrialized countries, they have even superseded diabetic retinopathy as the number one cause of blindness for people of working age [2,3]. They include heterogeneous forms of retinal dystrophies varying in their epidemiological, pathogenetic and clinical features [4,5,6]. Frequent initial symptoms are decreased vision, visual field defects, photophobia or nyctalopia [1,7]. While children often show severe courses, adults sometimes can preserve visual functions up to old age [7,8]. Nevertheless, severe vision loss up to complete blindness can occur in the final stages [1].

Over 270 genes have been linked to IRDs with different kinds of mutations and modes of transmission, potentially resulting in a similar clinical appearance [9]. For example, retinitis pigmentosa (RP), one of the commonest IRDs, is associated with over 130 genes [10]. On the other hand, mutations in the same genes can result in different IRDs. For instance, RP, cone–rod dystrophy (CRD), and Stargardt disease (STGD) may all occur associated with mutations of ABCA4 [10,11].

In general, a complete ophthalmological examination including fundus autofluorescence (FAF) and electroretinography (ERG) should reveal typical patterns of retinal dystrophies [1,12]. However, only molecular genetic analyses can identify the underlying gene mutations [1]. The pathogenesis of IRDs is potentially as variable as the responsible gene mutation. Globally, these defects result in a gradual decay of photoreceptors leading to visual impairment. For a long time, the distribution of lesions on a cellular level was poorly described due to a lack of histopathological material. Indeed, the in vivo analysis of structural changes occurring with retinal diseases has only been possible since the late 1990s, after optical coherence tomography (OCT) was invented in 1991 [13]. Today, OCT software programs provide three-dimensional visualization of the retina and estimations of quantitative parameters such as layer thicknesses. Still, the automatic segmentation of retinal layers becomes impossible in advanced cases of IRD when the continuity of retinal layers is affected [14,15,16]. Because manual segmentation is enormously time-consuming [15,17], many studies have only evaluated qualitative parameters such as the integrity of retinal layers, disruptions, or reflectivity [18,19,20,21,22]. Others have reduced the process of manual segmentation to a minimum by segmenting one or two foveal B-scans only or focussing on a single layer [11,23,24,25,26,27]. These approaches do not fully describe the three-dimensional structure of the retina or the local impact of IRDs, which may differ between the fovea and the periphery.

As a result, the present manuscript aims to provide a more comprehensive depiction of the retinal structure in genetically confirmed RP, CRD, and STGD by comparing manually segmented full macular SD-OCT scans to age and sex-matched controls. Parallel to the phenotypic disease entities, the impact of different genotypes on retinal layer changes was also investigated. Finally, interocular symmetry was assessed. 

## 2. Results

### 2.1. Alterations of the Retinal Structure in IRDs

Full macular volume scans obtained by SD-OCT were manually segmented and divided into nine subfields using the Early Treatment Diabetic Retinopathy Study (ETDRS) grid. The ETDRS grid consists of a central subfield within a 1 mm diameter from the fovea (C0), an inner ring within a 3 mm diameter from the fovea with four subfields (nasal (N1), temporal (T1), superior (S1) and inferior (I1)), and an outer ring within a 6 mm diameter from the fovea with another four subfields (nasal (N2), temporal (T2), superior (S2) and inferior (I2)). The resulting layer thicknesses were compared between disease groups and matched controls within each subfield using generalized least squares models. The demographic data are shown in Table 1. The genetic characteristics of all patients with an IRD are displayed in Table 2.

#### 2.1.1. Retinitis Pigmentosa

**Figure 1:**
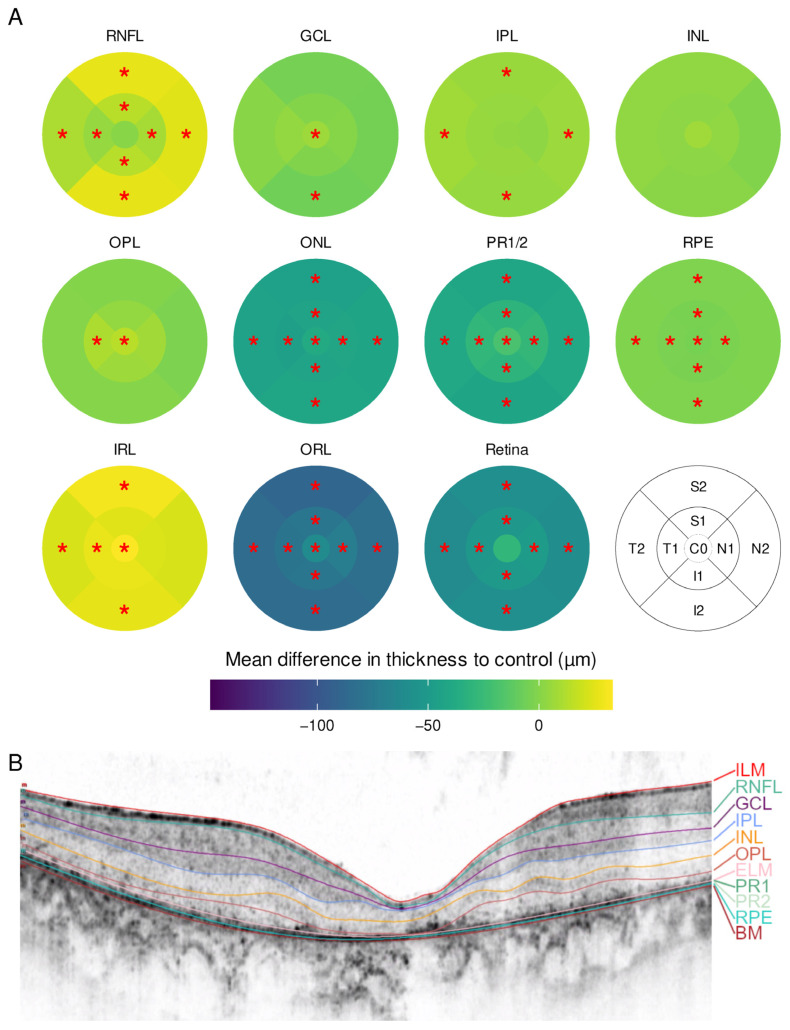
(**A**) Mean difference in layer thickness for Retinitis pigmentosa (RP) compared to controls represented as a heatmap on the ETDRS grid. Significant differences are marked with a red star (*) in the particular subfield. RNFL = retinal nerve fiber layer, GCL = ganglion cell layer, IPL = inner plexiform layer, INL = inner nuclear layer, OPL = outer plexiform layer, ONL = outer nuclear layer, PR1/2 = photoreceptor layer 1 and 2, RPE = retinal pigment epithelium, IRL = inner retinal layers (including RNFL, GCL, IPL, INL and OPL), ORL = outer retinal layers (including ONL, PR1/2, RPE); (**B**) example for a manually segmented SD-OCT scan from the fovea from RP patient 24.

Thirty-two eyes of sixteen patients with RP were compared to a sex- and age-matched group of individuals without retinal disease. Sixty-five subfields of different layers were significantly divergent between groups after *p*-value adjustment (Figure 1). Overall, RP resulted in a thinning of the retina while sparing the fovea. This was most obvious in the photoreceptor inner and outer segments (PR1/2) and the outer nuclear layer (ONL), where all subfields including C0 were affected. In contrast, the inner retinal layers (IRL) were thickened, either centrally (ganglion cell layer (GCL), outer plexiform layer (OPL)) or in the outer ring subfields (retinal nerve fiber layer (RNFL), inner plexiform layer (IPL)).

#### 2.1.2. Cone-Rod Dystrophy

**Figure 2:**
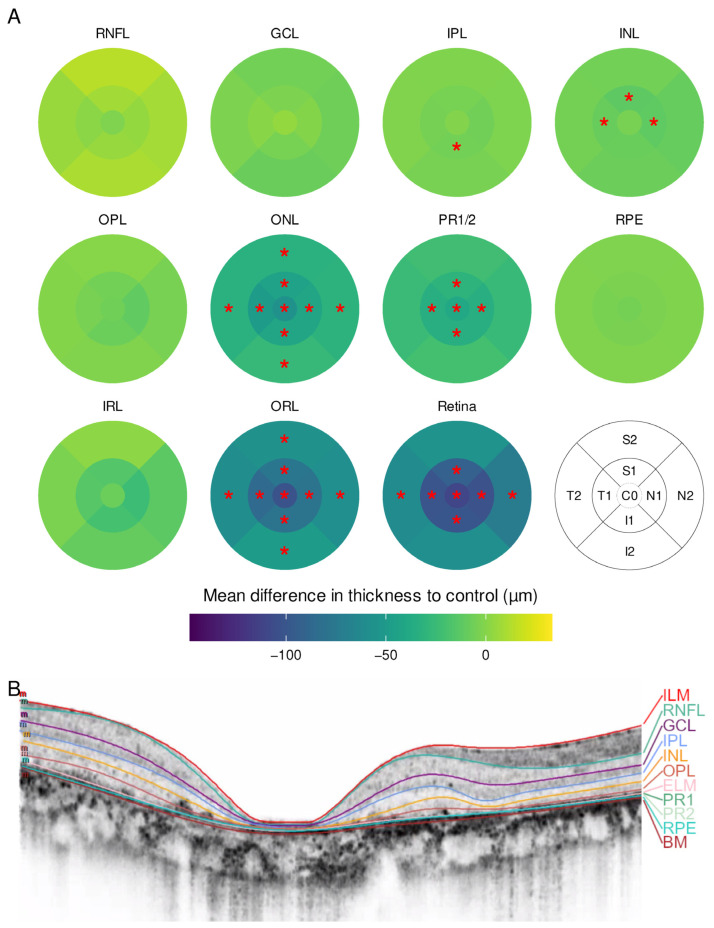
(**A**) Mean difference in layer thickness for cone–rod dystrophy (CRD) compared to controls represented as a heatmap on the ETDRS grid. Significant differences are marked with a red star (*) in the particular subfield; (**B**) example for a manually segmented SD-OCT scan from the fovea from CRD patient 5.

Fourteen eyes of seven patients with CRD were included. The sex- and age-matched control group consisted of seven individuals without retinal disease (Table 1). In the CRD group, a thinning of the whole retina was observed. There was no foveal sparing nor thickening of any subfield. In addition, the outer ring subfields were less impaired than the inner and central subfields. Again, ONL and PR1/2 were the most affected layers (Figure 2).

#### 2.1.3. Stargardt Disease

**Figure 3:**
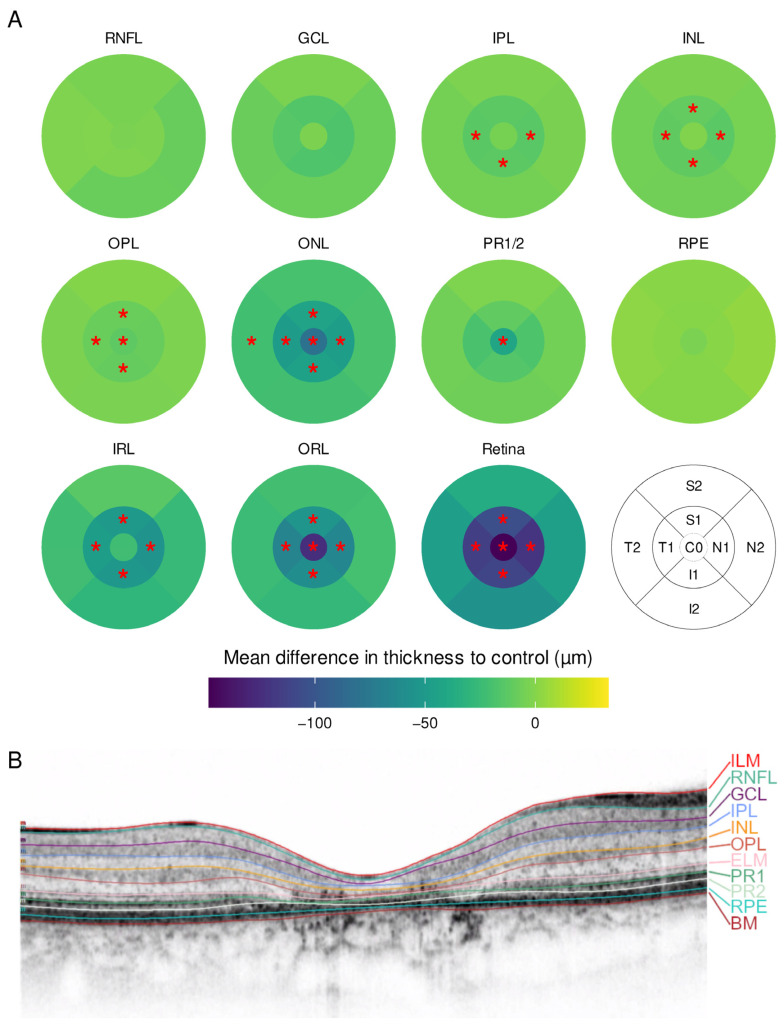
(**A**) Mean difference in layer thickness for Stargardt disease (STGD) compared to controls represented as a heatmap on the ETDRS grid. Significant differences are marked with a red star (*) in the particular subfield; (**B**) example for a manually segmented SD-OCT scan from the fovea from STGD patient 12.

Fourteen eyes of seven patients with Stargardt disease were included. They were compared to a group of seven individuals without retinal disease. The demographic information is summarized in Table 1. Patients with Stargardt disease displayed a similar pattern to CRD, with an overall thinning of the retina without foveal sparing (Figure 3). The thinning was predominantly observed in the ONL and OPL in the central subfield and inner ring as well as in the inner ring in IPL and inner nuclear layer (INL).

### 2.2. Association between Subfield Thicknesses and Visual Function

**Figure 4:**
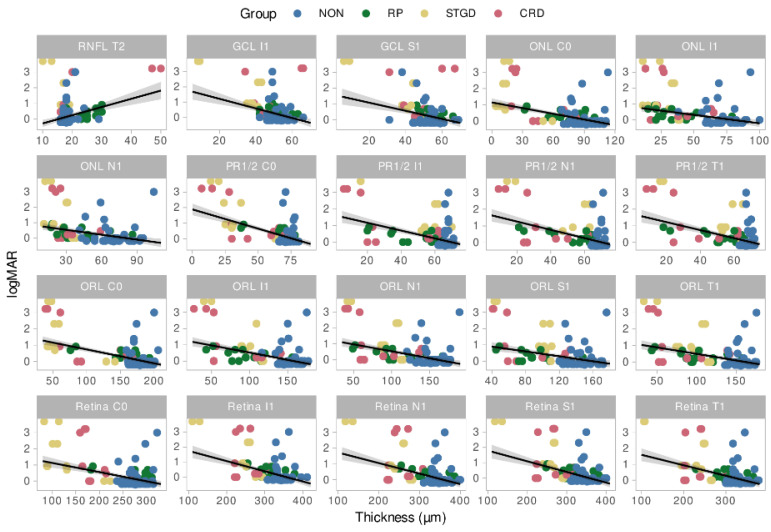
Relation between LogMAR VA and layer thickness for the 20 most significant subfields and layers. NON = Controls, STGD = Stargardt disease, RP = Retinitis pigmentosa, CRD = Cone rod dystrophy, Retina = Total retina thickness, RNFL = Retinal nerve fiber layer, ONL = Outer nuclear layer, PR1/2 = photoreceptor layer 1 and 2, GCL = ganglion cell layer, ORL = Outer retinal layers.

Decimal best-corrected Snellen VA was converted into the logarithm of the minimal angle of resolution (logMAR) for statistical analysis [28]. The logMAR VA was significantly higher in STGD (median: 0.916, z = 2.96, *p* = 0.009) and CRD (median: 0.693, z = 2.59, *p* = 0.03) compared to controls (median: 0) but not for RP (median: 0.343, z = 1.88, *p* = 0.164). Statistically significant relationships between visual acuity and layer thicknesses were observed in 34 subfields based on robust mixed linear models. The relation between logMAR VA and layer thicknesses for the 20 most significant subfields and layers is displayed in Figure 4. Overall, a negative correlation was found between logMAR and layer thickness for inner subfields of the whole retina (C0, I1, N1, T1), the outer retinal layers (in the subfields C0, I1, N1, T1 and S1), ONL (C0, I1, N1) and GCL (I1, S1), while the RNFL showed a positive correlation in T2.

### 2.3. Interocular Symmetry

**Figure 5:**
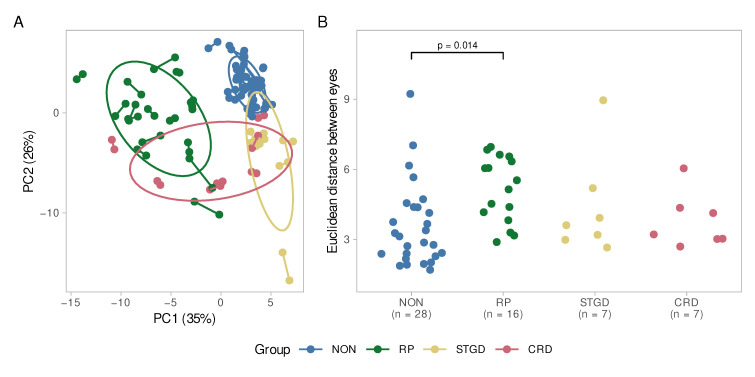
(**A**) Principal component analysis. The difference between both eyes of each patient is displayed. Each eye pair is connected by a line. The greater the distance between two points, the greater is the difference between the eyes of a patient. The ellipses represent the diseases as clusters. The controls (NON) and STGD group seem to be very homogeneously according to their small ellipses and short inter-eye distances. For the CRD group, there is a high interocular symmetry, while the intragroup variability is higher than in controls and STGD. The RP group reveals interocular as well as intragroup heterogeneity; (**B**) Euclidean distance between the eyes of the patients. Here, as well, the RP group shows a wider spread than the other diseases and the control group. The difference is significant compared to the control group (*p* = 0.014). NON = Controls, STGD = Stargardt disease, RP = Retinitis pigmentosa, CRD = cone–rod dystrophy.

In a principal component analysis, we can depict each eye as a point by summarizing all variables into fewer principal components capturing the bulk of the dataset’s variance. The distance between single eyes depends on their similarity. In Figure 5A, both eyes of each individual are linked. In general, the eyes of control individuals or patients with STGD or CRD are closer, indicating a higher level of symmetry than in patients with RP. The controls also appeared to form a dense cluster, while individuals with IRDs were more scattered, indicating a more variable profile of layer and subfield thicknesses. Similarly in Figure 5B, the Euclidean distance between both eyes of each individual was calculated. This value is a direct measure of the eyes’ similarity. The ANOVA indicated that there was an overall difference across groups (F(3, 53) = 3.03, *p* = 0.037; η^2^ = 0.15, 95% CI [0.005, 1.00]), which could be attributed to the higher intraindividual heterogeneity in patients with RP (*p* = 0.014) in the post-hoc test.

Patients with RP had the strongest interocular differences, mainly affecting the ONL (in all subfields), GCL (C0, T2, and I2), RNFL (C0, T1, and S2), OPL (I1 and I2) and retinal pigment epithelium (RPE, I2) with mean differences of 10–20%. However, this did not appear to impact total retina thickness.

It should be noted that the small sample sizes of STGD and CRD could possibly affect the significance of the results. In particular, the ONL seemed to be heterogeneously altered. For both groups, strong changes in the ONL thickness were visible in the inner ETDRS subfields C0, S1, N1 and I1. In STGD patients, a similar pattern was observed regarding the RPE and to some extent in the OPL and RNFL. 

The control group was not exempt from significant intraocular differences, for example in subfield C0 for the GCL, OPL and RPE as well as for S1 in the ONL and OPL and for S2 in the RNFL.

## 3. Discussion

The present study aimed to describe the changes in macular retinal layer thicknesses associated with RP, STGD and CRD. Most notably, we were able to report a diffuse thinning of the outer retinal layers in patients with RP sparing the fovea and partly compensated by an increase of the RNFL thickness. In CRD and STGD, the changes were slightly less pronounced and more symmetrical within patients. It appeared that CRD induces changes more locally in the central subfields, especially in the PR1/2 layer, while STGD predominantly affected the ONL.

As corroborated in this manuscript, retinal thinning was found in all subfields in RP patients. This is an essential feature of RP [29,30,31], which is mainly attributed to an atrophy of the outer retinal layers [30,32].

The ONL is mostly accountable for the ORL thinning. ONL thinning has been found repeatedly in humans and in animal models [23,33,34,35,36]. The degree of thinning is suggested to depend on disease stage and to be correlated with light sensitivity [23,34]. Hood et al. described a specific pattern in the transition zone in severely affected retinae, where a thinning of the photoreceptor outer subfields was followed by ONL thinning [32]. Although we were not able to confirm this order of changes, a strong dependence between these layers can be assumed.

Besides the ONL, the photoreceptor layer (PR1/2) was also strongly impacted in our RP group. The affection of this layer was described to eventually result in a thinning of the RPE [37,38].

Interestingly, we observed a thickening of the RNFL in all subfields with a stronger effect in the nasal, superior, and inferior than in the temporal region. Macular RNFL thickening is a common finding in other SD-OCT studies [30,34,39,40]. Nagasaka and Yoon used two meridian scans crossing the fovea vertically and horizontally. Supporting our results, their respective studies showed that the superior, nasal and inferior RNFL subfields were more thickened than the temporal side. Moreover, we found a slightly thickened RNFL in the superior and inferior subfields compared to the nasal one, which agrees with Nagasaka and Yoon’s data, although this pattern was not described previously. A thickened RNFL was also found peripapillary [41,42] next to thinned or normal RNFL [43,44], whereby the thickened subfield was mainly temporal. It should be noted that the temporal side of the optic nerve head directs to the nasal side of the macula. Thus, the observations of nasal macular and temporal peripapillary RNFL thickening blend perfectly into each other. However, the underlying pathology is still unclear. Possible reasons discussed are neuronal remodelling in response to the atrophic outer retinal layers, oedematous swelling or the proliferation of fibrous astrocytes [45,46]. Another interesting observation was made by Nagasaka et al. who found a relation between atrophic outer retinal layers, thickened inner retinal layers and an increased aqueous flare [30]. Thus, they suspect that inflammatory stimuli cause inner retinal thickening [30]. An interaction between RNFL thickening and GCL thinning was suggested by Hood et al. [40], who assumed a mechanical compensation of GCL loss by RNFL proliferation. In our observations, the patterns of RNFL and GCL fit in well; where the GCL is thinned, the RNFL is most thickened. Similar patterns can be suspected in the STGD and CRD groups. Nevertheless, as the effect is not significant, further investigations are required to prove an interaction between these layers.

It is worth noting that in previous studies the GCL was often measured in combination with the IPL [31,34,40]. Thus, the impact of the single layers remains uncertain. Nagasaka et al. found no changes in the IPL [30], but only within a 2 mm diameter of the fovea. Our measuring field of 6 mm diameter meanwhile revealed a thickening of all outer ring subfields. Consequently, combining the GCL and IPL may result in an underestimation of GCL thinning and overestimation of GCL thickening while concealing the relation to the RNFL. 

Latest investigations on a mouse model demonstrated a hypertrophy of the horizontal cells in the INL of the central retina [47]. Under the assumption that hypertrophic processes lead to a thickening of the corresponding layer, our results provide a clinical correlate to this observation with a central INL thickening. However, these alterations remain minimal, providing INL cell types as a therapeutic target.

To our knowledge, this is the first study describing the OPL as a single layer in SD-OCT for IRDs. Interestingly, there is a tendency of thinning in the outer ring areas while the central and inner ring areas are thickened. The loss of connecting fibers due to dendritic retraction, mainly of rod bipolar cells, in the OPL has been described previously and can explain our findings of outer ring thinning [37,47]. In contrast, in an ultrastructural analysis, Stefanov et al. reported hypertrophy of amacrine cell processes in the OPL for the central retina, suggesting that this would cause central retinal thickening [47]. Our findings support this assumption.

In cone–rod dystrophies, cones are primarily affected in a centrifugal way. We could observe retinal thinning in the central and inner ring subfields, particularly affecting the ONL, where it even extended into the outer temporal subfield. This confirms previous OCT and histopathological results [48,49,50].

Altogether, there is a lack of data for retinal layer thicknesses other than the ONL and RNFL. In the outer retinal layers, ONL and PR1/2, the central pattern was similar to the one found in RP patients. This is also consistent with previous findings [22,48,49]. It must be noted that these studies examined layer thicknesses only centrally or in a 2 mm diameter around the fovea centralis. In contrast, although we could not find significant differences in thinning or thickening in the RPE, other authors described both effects [48,49]. Although larger cohorts of respectively fifteen and sixteen patients were examined by Cho and Zahlava, they segmented fewer B-scans (one foveal B-scan and six B-scans), which was shown to be a potential source of bias [15].

We found a tendency for a slightly thicker RNFL in the superior and inferior outer subfields. In contrast to our findings, another study described peripapillary RNFL thinning in the temporal, macula-directing quadrant [40]. The precisely opposite patterns of RNFL and GCL in CRD would fit Hood and colleagues’ assumption in RP patients. However, these changes were not significant, which could result from the small sample size. 

Overall, the inner retinal layers appeared to be more preserved than the outer retinal layers as it occurs in RP. It is unclear whether these layers may become affected in later disease stages. 

As described previously [26], STGD was associated with a centrifugal thinning in all sublayers. As in our study, the ONL was most severely affected.

In the present cohort, the RNFL showed a significant central thinning. Previous studies reported controversial findings regarding RNFL thickness changes in STGD. While some authors found a thinned macular or peripapillary RNFL [26,51,52], the most extensive study to date revealed thickening of the RNFL [53]. Interestingly, Genead and colleagues found a thinned peripapillary RNFL in half of their patients, while a few (18.5%) showed thickening [54]. In summary, the RNFL may be variably affected by STGD, and this effect also depends on disease progression.

LogMAR visual acuity was significantly associated with the thicknesses of retinal layers in various subfields. As described previously, these associations were generally positive, meaning that thicker retinal layers were associated with better visual acuity. This is consistent with previously reported studies [26,31,34,55,56]. However, a negative correlation was found for the RNFL, which corroborates Yoon et al.’s observations in patients with RP [39]. The most affected subfield was C0, representing the foveal region. Thus, we can confirm a significant relationship between visual acuity and the integrity of retinal structures. 

Macular dystrophies are often described as bilateral diseases [57,58,59], but there are only a few studies investigating for interocular symmetry. In the STGD and CRD groups, we found a high degree of interocular symmetry which corroborates former results based mainly on BCVA, ERG, FAF or perimetry [24,60,61]. In contrast, the RP group showed a more pronounced asymmetry which contradicts results obtained by ERG, perimetry and FAF [62,63]. Even with SD-OCT, Tee et al. found no difference in the rate of ONL thinning between eyes [64]. One possible explanation for these differences may be that the lesions induced by RP are distributed more randomly on the retina than in other retinal dystrophies. This would result in interocular asymmetry with similar functional impairment, which is more readily assessed with ERG. We may speculate that the changes in retinal layer morphology, although large enough to indicate statistically significant asymmetry, are too small to result in clinical differences in ERG, perimetry or FAF. Moreover, various studies investigated different biomarkers concerning symmetry and, to our knowledge, this study was the first to include different retinal layer thicknesses. However, asymmetry was only obvious when facing single retinal layers in contrast to the total retina thickness which, considered alone, would have suggested a high rate of interocular symmetry. 

In light of the present findings, the bilateral assessment of retinal lesions is especially important in patients with RP.

## 4. Materials and Methods

### 4.1. Participants

This is a retrospective study conducted in adherence to the tenets of the Declaration of Helsinki. The study protocol was approved by the Ethics committee of Hannover Medical School (Nr. 9426_BO_S_2020). Written consent was obtained from all patients or a legal guardian.

Patients with genetically confirmed RP, STGD, or CRD presenting at the department of ophthalmology from Hannover Medical School between 2014 and 2019 were included in the study. Age- and sex-matched controls without retinal pathologies were selected alongside. Age matching was performed within age groups spanning five years (i.e., 10–15 years of age; 15–20 years of age, etc.). Some control patients were matched to several patients with different inherited retinal diseases. All participants underwent a complete ophthalmological examination, including refraction, best corrected Snellen visual acuity converted to the logarithm of the minimum angle of resolution (logMAR), slit lamp biomicroscopy, applanation tonometry, fundus examination, SD-OCT, fundus autofluorescence, multifocal and full-field electroretinogram, as well as genetic testing. 

### 4.2. Molecular Genetic Analysis

Blood samples of all included patients were taken for DNA extraction after informed consent had been signed according to the German Genetic Diagnostics Act. Molecular genetic testing was done by Next Generation Sequencing (NGS) using targeted gene panels [65,66]. Classification of variants followed the recommendations by the American College of Medical Genetics and Genomics (ACMG) standards and guidelines and the Association for Molecular Pathology (AMP) Clinical Practice Guidelines [67].

### 4.3. Imaging and Segmentation Using SD-OCT

Macular volume scans were captured using SD-OCT (Spectralis OCT, Heidelberg Engineering, Heidelberg, Germany) by qualified photographers. The full macular scans consisted of 49 horizontal, equidistant (124 µm) linear B-scans in a 20 × 20° area centered on the fovea. Scans of poor quality were excluded due to insufficient fixation or opacity of the optical axis (lens, cornea, or vitreous body). The macula scans were divided into nine subfields using the Early Treatment Diabetic Retinopathy Study (ETDRS) grid with three rings at 1, 3 and 6 mm diameter and four radial lines (C0, N1, N2, I1, I2, T1, T2, S1 and S2). The ETDRS grid was manually centered on the anatomic fovea centralis. In four cases, single peripheral subfields were outside the detected OCT scan area after centering due to poor fixation of the patients. An example is displayed in the Supplementary Figure S3B, where the I2 subfield could not be measured.

Automated segmentation from the Heidelberg Eye Explorer software (version 6.7.13.0, Heidelberg Engineering GmbH, Heidelberg, Germany) was confirmed or corrected manually by an experienced grader and then reviewed by another experienced grader. It consisted of the identification of the internal limiting membrane (ILM), the border between the RNFL and the GCL, the border between the GCL and the IPL, the border between the IPL and the INL, the border between the INL and the OPL, the border between the OPL and the ONL, the external limiting membrane (ELM), the border between the PR1/2 and the RPE, and of Bruch’s membrane (BM).

At least 45 out of 49 B-scans were manually segmented when automated segmentation failed. The process of manual segmentation took one to four hours per eye. If the image quality of single scans was too poor to distinguish single layers, their boundaries were set based on the adjacent B-scans. In atrophic zones, the layers were superimposed right upon each other. Examples for segmented SD-OCT B-scans are shown in Figure 1B, Figure 2B and Figure 3B. Additional SD-OCT data for exemplary patients as well as for one control is displayed in the Supplementary Figures S1–S4.

From these borders, the mean thickness (in µm) of the RNFL (as distance between ILM and RNFL border), GCL (as distance between RNFL and GCL borders), IPL (distance between GCL and IPL borders), INL (distance between IPL and INL borders), OPL (distance between INL and OPL borders), ONL (distance between OPL and ONL borders), ELM (distance between ONL border and ELM), PR1/2 (distance between ELM and RPE border) and RPE (distance between RPE and BM), the IRL (as the distance between ILM and OPL/ONL border), the ORL (as the distance between OPL/ONL border and BM) and the total retina (as the distance between ILM and BM) could be obtained for each ETDRS subfield and exported to a spreadsheet for statistical analysis.

### 4.4. Statistical Analyses

The statistical analysis was performed with R version 4.2.1 [68]. For each disease, the thickness of every subfield within each layer was compared to matched controls using generalized least squares models accounting for repeated measures (correlation within each patient) and heteroscedasticity (disease-specific constant variance) using the ‘nlme’ R-package [69,70]. The regression coefficients corresponding to the mean difference in layer thickness between patients and controls were depicted as heatmaps reproducing the ETDRS grid. The color scales of the heatmaps were harmonized to allow for comparison between figures.

Using the ‘robustlmm’ R-package [71], robust mixed linear models with patient as a random variable were fitted to assess the relationship of vision (logMAR) and layer thickness. 

In both cases, the *p*-values were computed using a Wald *t*-distribution approximation as implemented in the ‘parameters’ R-package [72] and adjusted for multiple comparisons using the Holm procedure [73].

As logMAR did not satisfy distributional assumptions for a parametric test, it was compared across groups using the Kruskal–Wallis tests. For significant results, Dunn-tests were used subsequently to compare the individual IRDs to the control group. The *p*-values were adjusted based on a multivariate normal distribution as implemented in the ‘PMCMRplus’ R-package [74]. 

To assess interocular symmetry, the Euclidean distance between both eyes of each individual were computed using the scaled subfield thicknesses, so that thicker subfields do not overshadow thinner ones. These values were compared across groups with a one-way ANOVA. Post-hoc comparisons were performed using the ‘emmeans’ R-package and Dunnett-type contrasts with the corresponding *p*-value adjustment [75]. Since each control was matched to one or more patients with retinal disease, there are more controls than patients within each group but fewer controls than patients overall. Therefore, the groups in the symmetry analysis were of unequal size.

Additionally, interocular symmetry was depicted in a principal component analysis computed using the ‘prcomp’ R-function on the scaled and centered data after removal of summary values (i.e., the IRL, ORL, and retina thickness) and imputation of missing values (109/12177, 0.9%) using the *k*-nearest neighbors algorithm, as implemented in the ‘impute’ R-package [76]. In addition, the percent difference between the thickness of each subfield and layer was calculated and analyzed using a one-sided Wilcoxon signed rank test of the null that the difference between sides is smaller than 5%. The *p*-values were adjusted using the Holm procedure within group and layer. The results are presented as a heatmap of mean relative differences between sides.

In every case, model residuals were inspected visually, and statistical significance was accepted at *p* ≤ 0.05 (after correction for multiplicity).

## 5. Conclusions

Despite the low prevalence of IRDs resulting in small cohorts, the present study aimed to provide an overview of the retinal changes induced by RP, STGD, and CRD in a comparative approach backed by extensive manual segmentation of SD-OCT scans. These revealed thinned outer subfields for RP and thinned central subfields for CRD and STGD. The ONL and photoreceptor layers were most affected and may provide biomarkers for IRDs. We could demonstrate that, although bilateral, RP is less symmetric than CRD or STGD. Young or strongly visually impaired patients remain a challenge in OCT studies due to poor fixation. Overall, this study confirms assumptions about retinal remodeling in IRDs previously based on single cross-sectional SD-OCT images of the fovea using a more comprehensive characterization of the whole retina. These results are relevant for the development of specific therapeutic approaches. Moreover, the suggested structural biomarkers may enable to distinguish between disease entities early on. Longitudinal studies are warranted to describe the temporal component of those changes and better assess disease progression. 

## Figures and Tables

**Table 1:** Demographic information for RP, STGD, and CRD patients and controls.

	Retinitis Pigmentosa		Stargardt Disease		Cone-Rod Dystrophy	
	Controls (*n* = 16)	RP (*n* = 16)	Controls (*n* = 7)	STGD (*n* = 7)	Controls (*n* = 7)	CRD (*n* = 7)
Age						
Mean (SD)	37.0 (15.2)	36.7 (15.7)	40.0 (18.4)	40.3 (17.2)	34.7 (11.8)	33.4 (10.8)
Median	39.3	39.2	37.5	37.2	36.2	36.0
[Min, Max]	[8.09, 59.0]	[7.95, 60.0]	[10.8, 70.2]	[14.1, 69.8]	[17.7, 52.3]	[18.4, 49.5]
Sex (*n*)						
Female	8	8	5	5	3	3
Male	8	8	2	2	4	4

**Table 2:** Genetic data of all IRD patients.

Disease	Patient ID	Family ID	Age	Sex	Gene	Sequence Variant	Status
CRD	1	1	49.5	m	*ABCA4*	c.4615_4625del, c.5603A>T	heterozygous
	2	2	18.4	f	*ABCA4*	c.5917del	homozygous
	3	3	24	f	*ABCA4*	c.1903C>T c.5882G>A	heterozygous
	4	4	40.1	f	*CACNA2D4*	c.2854C>T	heterozygous
	5	5	26.9	m	*ABCA4*	c.5917del	homozygous
	6	6	36	m	*RPGR*	c.3070G>T	hemizygous
	7	7	38.9	m	*ABCA4*	c.[1622T>C(;)3113C>T], c.5714+5G>A	heterozygous
STGD	8	8	37.2	f	*ABCA4*	c.5882G>A, c.5381C>A	heterozygous
	9	8	69.8	f	*ABCA4*	c.5381C>A	homozygous
	10	9	35.3	f	*ABCA4*	c.5582G>A, c.1609C>T	heterozygous
	11	10	50.6	m	*ABCA4*	c.5882G>A	homozygous
	12	11	42.4	m	*ABCA4*	c.67-1G>C, c.2804T>C	heterozygous
	13	12	32.6	f	*ABCA4*	c.4849-2A>G, c.5882G>A	heterozygous
	14	13	14.1	f	*ABCA4*	c.[1609C>T;5881G>A]	homozygous
RP	15	14	35.6	f	*PRPH2*	c.646C>T	heterozygous
	16	15	30	m	*RP1*	c.662del	homozygous
	17	16	60	m	*NR2E3*	c.166G>A	heterozygous
	18	17	48.1	f	*USH2A*	c.3551T>A, c.14131C>T	heterozygous
	19	18	41	m	*EYS*	deletion Exon 12	homozygous
	20	19	37.4	m	*PRPF31*	c.1060C>T	heterozygous
	21	20	52.2	m	*USH2A*	c.[2299del(;)4714C>T], c.2276G>T	heterozygous
	22	21	24	f	*PCARE*	c.1541del	homozygous
	23	22	48.6	f	*RP1*	c.2332A>T	heterozygous
	24	23	56.2	f	*FLVCR1*	c.1092+5G>A	homozygous
	25	24	43.8	f	*PCARE*	c.1541del	homozygous
	26	25	8.34	f	*USH2A*	c.2299del	heterozygous
	27	26	7.95	m	*CDH23*	c.2206C>T, c.6000C>A	heterozygous
	28	27	35	m	*USH2A*	c.1876C>T, c.11864G>A	heterozygous
	29	28	18.2	f	*NPHP1*	deletion Exon 1-20	homozygous
	30	29	41.2	m	*USH2A*	c.2276G>T, c.10010G>T	heterozygous

## Data Availability

The datasets used and/or analyzed during the current study are available from the corresponding author on reasonable request.

## Supplementary Materials

The following supporting information can be downloaded at: https://www.mdpi.com/article/10.3390/ijms232416007/s1.
