# Supplementary material for: Investigation of Structural Alterations in Inherited Retinal Diseases: A Quantitative SD-OCT-Analysis of Retinal Layer Thicknesses in Light of Underlying Genetic Mutations
Source: Int J Mol Sci. 2022 Dec 16;23(24):16007. doi: 10.3390/ijms232416007 (PMC9788460; doi:10.3390/ijms232416007)
Supplement: Supplementary file 1 [file ijms-23-16007-s001.zip › ijms-2065634-supplementary.pdf]

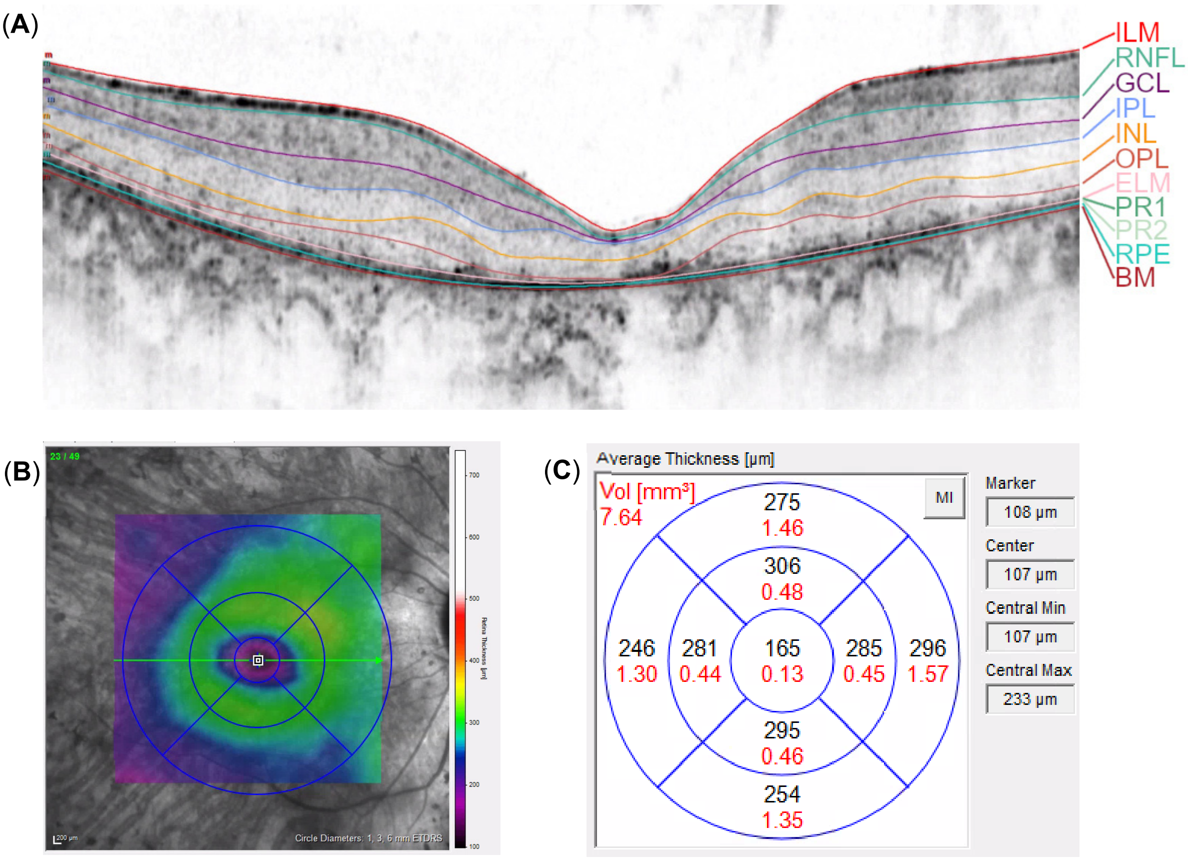

Figure S1. (A) Example for a manually segmented SD-OCT scan from the fovea from Retinitis pigmentosa patient 24. RNFL = retinal nerve fiber layer, GCL = ganglion cell layer, IPL = inner plexiform layer, INL = inner nuclear layer, OPL = outer plexiform layer, ONL = outer nuclear layer, PR1/2 = photoreceptor layer 1 and 2, RPE = retinal pigment epithelium, IRL = inner retinal layers (including RNFL, GCL, IPL, INL and OPL), ORL = outer retinal layers (including ONL, PR1/2, RPE). **(B)** Corresponding Early Treatment Diabetic Retinopathy Study (ETDRS) grid to Figure S1A. Green line = B-scan that is displayed in Figure S1A. **(C)** Corresponding ETDRS grid with average thickness measurements in µm (black) and volume measurements in mm^3 (red).

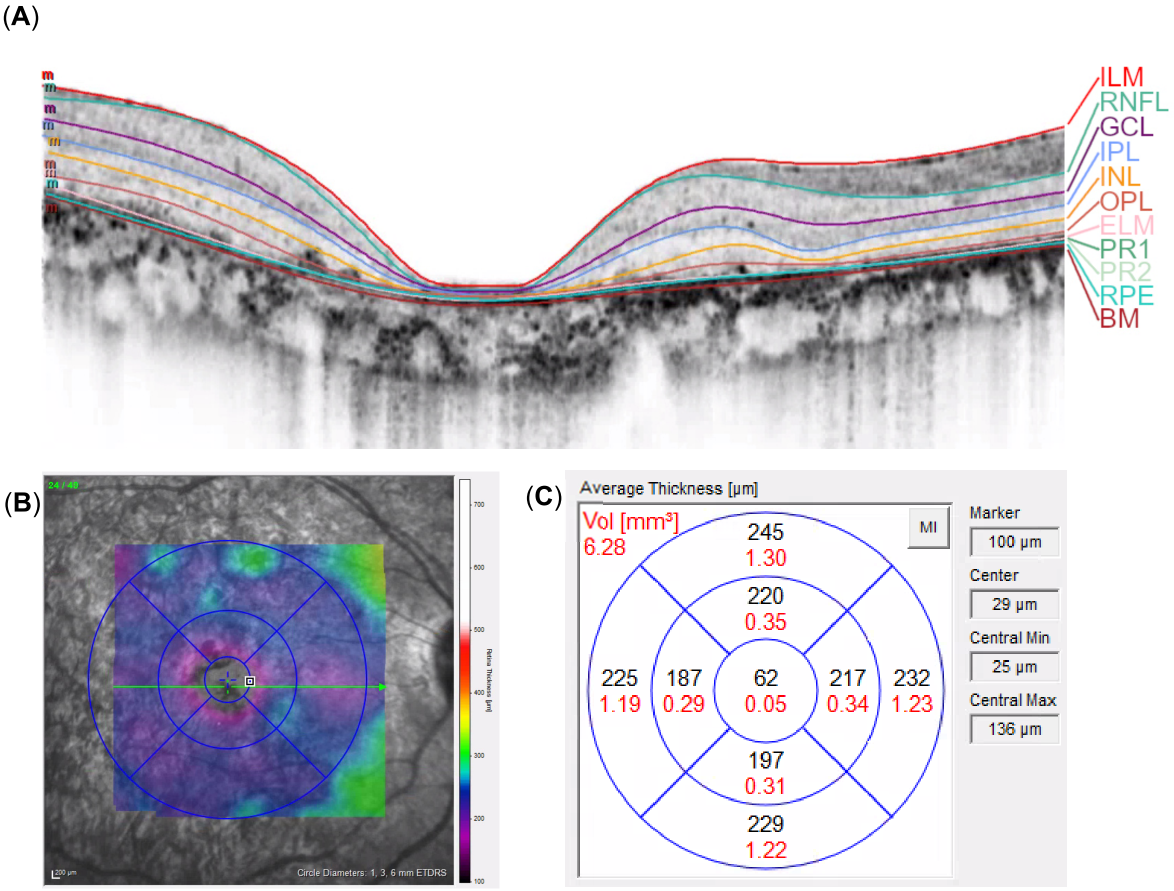

Figure S2. (A) Example for a manually segmented SD-OCT scan from the fovea from Cone-rod dystrophy patient 5. **(B)** Corresponding ETDRS grid to Figure S2A. Green line = B-scan that is displayed in Figure S2A. **(C)** Corresponding ETDRS grid with average thickness measurements in µm (black) and volume measurements in mm^3 (red).

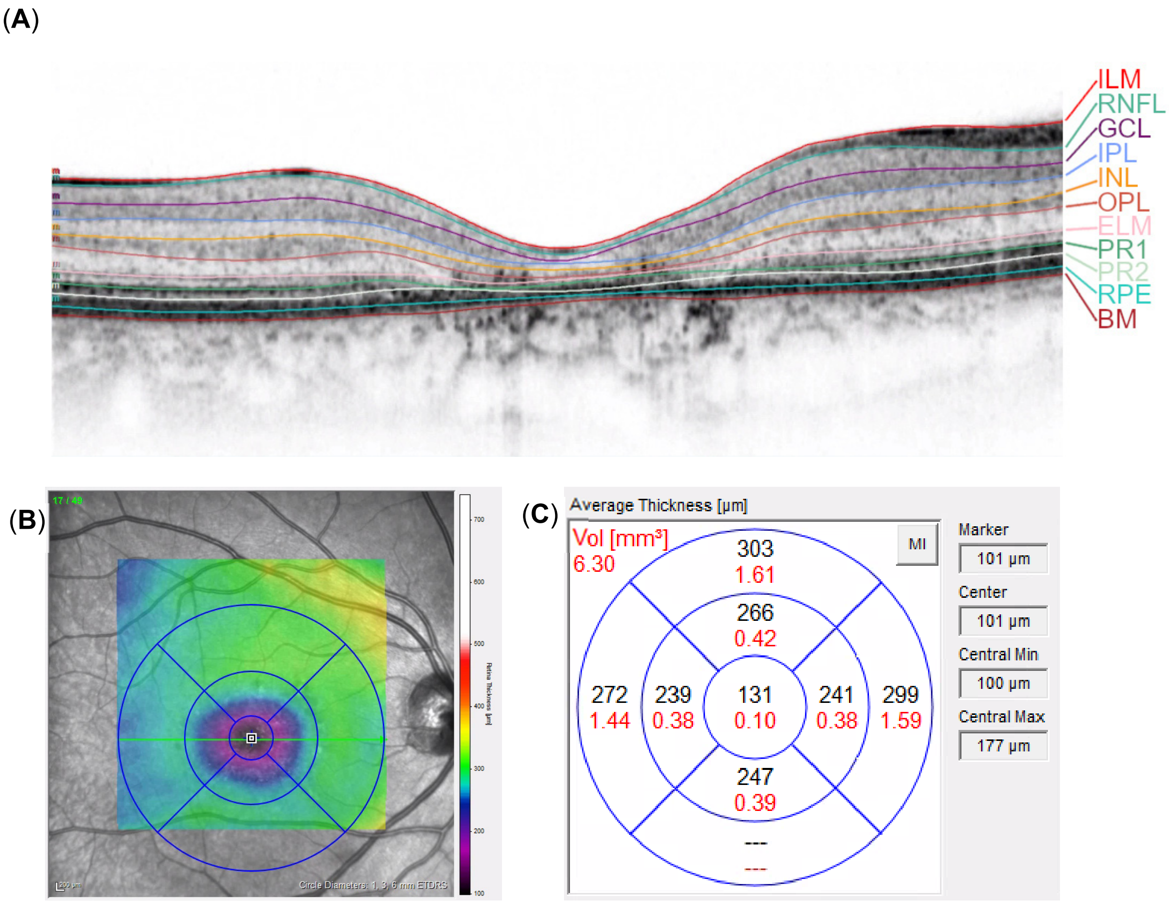

Figure S3. (A) Example for a manually segmented SD-OCT scan from the fovea from Stargardt disease patient 5. **(B)** Corresponding ETDRS grid to Figure S3A. Green line = B-scan that is displayed in Figure S3A. **(C)** Corresponding ETDRS grid with average thickness measurements in µm (black) and volume measurements in mm³ (red).

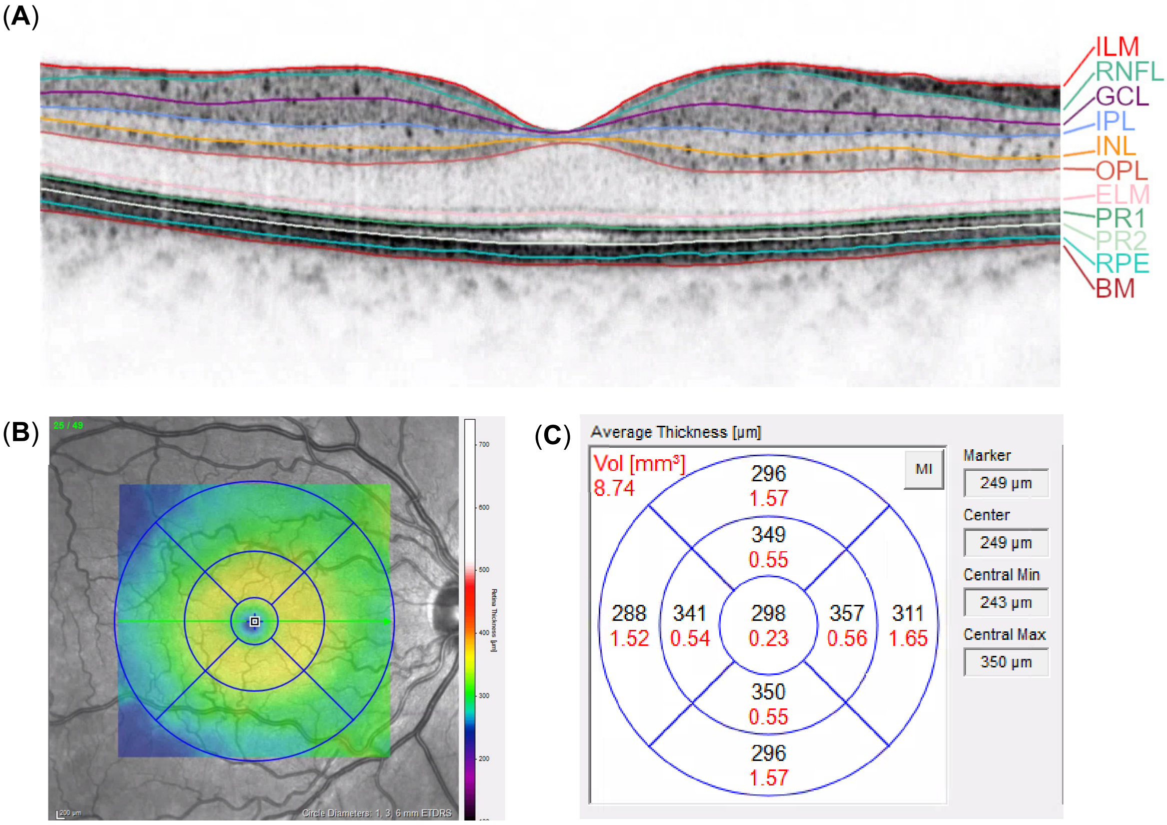

Figure S4. **(A)** Example for a manually segmented SD-OCT scan from a control patient. **(B)** Corresponding ETDRS grid to Figure S4A. Green line = B-scan that is displayed in Figure S4A. **(C)** Corresponding ETDRS grid with average thickness measurements in µm (black) and volume measurements in mm^3 (red).
